# Clinical presentations of substance abuse in bipolar heroin addicts at time of treatment entry

**DOI:** 10.1186/1744-859X-11-23

**Published:** 2012-09-03

**Authors:** Icro Maremmani, Angelo Giovanni Icro Maremmani, Fabio Rugani, Luca Rovai, Matteo Pacini, Silvia Bacciardi, Joseph Deltito, Liliana Dell’Osso, Hagop S Akiskal

**Affiliations:** 1 Vincent P. Dole Dual Diagnosis Unit, Santa Chiara University Hospital, Department of Neurosciences, University of Pisa, Pisa, EU, Italy; 2 Association for the Application of Neuroscientific Knowledge to Social Aims (AU-CNS), Pietrasanta, Lucca, EU, Italy; 3 G. De Lisio Institute of Behavioural Sciences, Pisa, EU, Italy; 4 Department of Psychiatry and Behavioural Sciences, New York Medical College, Valhalla, NY, USA; 5 International Mood Centre, University of California, La Jolla, CA, USA

## Abstract

**Background:**

Studies on the ‘self-medication hypothesis’ have focused on substance abuse as an attempt to alleviate emotional suffering.

**Methods:**

We have investigated concomitant substances of abuse in 150 bipolar heroin addicts clustered according to their clinical presentation at treatment entry (depressive episode, hypomanic episode, manic episode and mixed episode). Bipolar heroin addicted patients were chosen because they tend to have a concomitant poly-substance abuse and because, as compared with patients suffering for other mental illnesses, they more clearly reveal a variety of identifiable affective states.

**Results:**

Patients with a depressive episode more frequently used non-prescribed anxiolytic-hypnotics. They were found to use cocaine-amphetamines more frequently during a hypomanic episode, whereas the use of cannabis and cocaine-amphetamines occurred more frequently during a manic episode. The associated use of alcohol, cocaine-amphetamines and cannabinoids was more frequently encountered during a mixed episode. Limitations: apart from the difficulty in determining whether the substance use modifies the mood or the mood state determines the substance used, this is a report on a retrospective analysis, rather than a study specifically designed to elucidate the issue; in addition, no information was available on the temperament of our subjects. Assessments of the same subject in various clinical presentations would have provided a better level of information.

**Conclusions:**

Besides one expected result – the prominent use of CNS stimulants during a depressive phase of bipolar patients – this study supports the hypothesis that mood elation is a pleasurable, rewarding experience that, in bipolar patients, can be started or prolonged by means of CNS stimulant drugs. Stimulant use was, therefore, more prevalent during the ‘up’ rather than the ‘down’ phase of the illness.

## Background

Psychiatric disorders and substance abuse commonly co-occur. Population-based studies have provided documentation that, of all patients with major psychiatric disorders, those with bipolar disorder show the highest prevalence of comorbid substance abuse and dependence. The cause of this high comorbidity rate has not been clearly established, and the relationship is probably bidirectional [1]. One explanation for this co-occurrence is the 'self-medication hypothesis', which states that some patients experience improvement in psychiatric symptoms as a result of substance use [2].

It has been found that about 50% of individuals with bipolar disorder have a lifetime history of substance abuse or dependence [3]. Furthermore, bipolar I subjects appear to have higher rates of these comorbid conditions than bipolar II subjects [4].

Research has consistently shown that substance abuse in bipolar patients may have negative consequences both on clinical characteristics and long-term course: drug addiction is associated with medication non-compliance [5], a higher frequency of mixed or dysphoric mania and, possibly, an earlier onset of affective symptoms [6,7], more severe impairment of social functioning [8], greater subjective distress and less resourcefulness in coping [9], more hospitalizations and poorer prognoses [10,11], together with a higher frequency of suicide attempts [12,13].

Alcohol and cannabis are the substances most often abused, followed by cocaine and then opioids. In terms of specificity, a link seems to exist between cocaine use, as evaluated among poly-abusers of different categories, and bipolar disorders [14-16]. When abusing cocaine, bipolar patients showed significantly higher rates of post-traumatic stress disorder and antisocial personality disorder, and were more likely to present in a mixed mood state [17].

Alcohol abuse and dependence show a lifetime prevalence 3–4 times higher in patients with bipolar disorders than in the general population, while the lifetime prevalence of mood disorders in alcohol-dependent subjects is approximately 10 times higher than in the general population [18]. Most bipolar patients run the risk of developing lifetime drug or alcohol-related problems, which may in their turn contribute to more varied and complex clinical presentations, so increasing the risk of a depressive episode in the near term [19], poorer lithium response, functional disability and elevated suicide risk [20], as well as high rates of suicide attempts [21,22]. Moreover, alcohol addiction may exacerbate impulsive behaviours and risk-taking propensities in bipolar patients [23].

With respect to cannabis use, some papers have pointed out that marijuana is not only often abused by patients suffering from bipolar disorder, but also induces manic symptoms [24-27]. Additionally, cannabis-using bipolar patients experienced less satisfaction with life, had a lower probability of having a romantic relationship compared with non-users [28,29], and also had more severe alcohol and other drug use [30].

Regarding heroin use, very few studies have been conducted on the specific effects of its abuse in the clinical course of bipolar patients [16,31].

Studies on the self-medication hypothesis (SMH) have focused on the use of heroin and cocaine dependence as an attempt to alleviate emotional suffering [32]. Most addicts do not choose drugs randomly to alleviate painful affective states and their underlying psychiatric disorders. Rather, drugs are chosen because an individual discovers a specific psychopharmacological action that helps to alleviate an individual’s suffering. Recently, greater emphasis has been placed on understanding addiction as a form of ‘self-medication’ to alleviate suffering, with less emphasis on its severe psychopathology.

As the ‘self-medication’ theory suggests, patients to modulate their mood by decreasing their dysphoria may use heroin. In other words, patients appear to select substances that they expect to have a ‘healing’ effect. In bipolar patients cocaine appears to exercise an appeal because of its ability to relieve distress associated with depression [32]. Recently Khantzian revised his theory [33] and expanded the number of affective states to be examined, including alexithymia, to better operationalize SMH, but some authors indicated that affective measures did not have the expected relationship with reported substance use [34,35]. In this study, to further test the validity of Khantzian’s hypothesis, we compared concomitant substances of abuse in bipolar heroin addicts according to their clinical presentation (depressive episode, hypomanic episode, manic episode and mixed episode). Bipolar patients have been chosen because, as compared with patients suffering for other mental illnesses, they are more likely to clearly show various different identifiable affective states. We considered heroin-dependent bipolar patients as Khantzian developed his hypothesis treating heroin addicts. Moreover, bipolar patients are generally multi-drug abusers. The choice of these patients is also interesting for the fact that heroin use in bipolar disorder is perhaps the least understood and researched groups of patients with bipolar disorder and substance use.

Khantzian’s hypothesis would be supported if the use of CNS stimulants were prominent in the depressive phase and CNS depressants in hypomanic or manic phases, at least when patients complain of altered mood or insufficient balance of affective symptoms despite putative self-medication by substance use.

## Methods

### Design of the study

This is a retrospective, observational, case–control study. The research study was implemented using a dataset from previous studies on MMTP carried out in Italy and used in previous published articles (Pisa addiction dataset: a database including anonymous individual information originally collected for clinical and research purposes).

The study included patients treated at Santa Chiara University Hospital, Department of Psychiatry, University of Pisa, Italy during the period 1994–2010. All patients gave their informed consent to the anonymous use of their personal data records for research purposes.

### Sample

Patients included in the Pisa addiction dataset were selected on the basis of the following characteristics:

Diagnosis of Bipolar I or II disorders according to DSM-IV TR and various DSM criteria

Diagnosis of opioid dependence with physical dependence according to DSM-IV TR and various DSM criteria

Being at their lifetime first hospitalization

Not receiving medication for the treatment of bipolar disorders

Using at least two substances of abuse (heroin included) at study entry

We excluded patients:

Receiving opioid agonist medications for opioid dependence

Using illegal methadone

Patients using opioid agonist medications or illegally obtained methadone were excluded to limit the confusing effects of this medication on clinical psychiatric presentation [36].

We included in the study 150 consecutive bipolar heroin addicts. The mean age of these patients was 29 ± 6 years old (range 17–50). Ninety-one (60.7%) were male; 85 (56.7%) were single, 104 (69.3%) had less than 9 years of education, and 70 (46.7%) were unemployed.

As to drug addiction history, 127 (84.7%) patients reported physical complications. 100 (66.7%) were unemployed, 92 (61.3%) showed household, 53 (35.3%) romantic, 81 (54.0%) social leisure and 72 (48.0%) legal difficulties. 86 (57.3%) were poly-abusers (3 or more substances), 120 (80.0%) had experienced past treatment failures.

In order to obtain groups of patients differing in clinical presentation at hospitalization, patients were divided into four groups on the basis of their episode polarity. 103 patients (68.6%) showed a major depressive episode; 22 (14.7%) a hypomanic episode; 5 (3.3%), a full manic episode and 20 (13.3%) a mixed episode, according DSM-IV TR and various DSM criteria.

### Instruments

#### Drug addiction history questionnaire (DAH-Q)

Addiction-related information was collected by means of DAH-Q [37] administered by a psychiatrist. The DAH-Q is a multi-scale questionnaire that comprises the following categories: demographic data, physical health (hepatic, vascular and lymphatic pathology, gastrointestinal disorders, sexual disorders, dental pathology, HIV seropositivity), mental health (awareness of illness, memory disorders, anxiety disorders, mood disorders, aggression, thought disorders, sensory perception disorders), substance abuse (use of alcohol, opiates, CNS depressants, CNS stimulants, hallucinogens, phencyclidine, cannabis, inhalants, poly-substance abuse), social adjustment and environmental factors (employment family, sex, socialization and leisure time, legal problems), clinical characteristics as frequency of drug use, patterns of use, phase, nosology, treatment history (previous and current treatments). Items are set up so as to elicit dichotomous answers (yes/no).

#### Substance Use

Regarding toxicological urinalyses, we utilized the routine analyses as used for all hospitalized patients. The enzyme-multiplied immunotechniques for opiates, methadone, benzodiazepines, hypnotics, cocaine, amphetamines, hallucinogens, cannabinoids and inhalants were used. Problematic alcohol use was defined according to a lifetime history of frequent intoxication and/or negative consequences of habitual use on their social adjustment (work, family, social/leisure or legal issues).

#### Data analysis

The 4 groups clustered according to the clinical presentation at hospitalization were compared for demographic, DAH-Q factors and concomitant substance abuse by means of the chi-square test for categorical variables (with contrasts), and one-way analysis of variance for continuous variables, a posteriori contrasts according to the Scheffe’s procedure.

All analyses were carried out using the statistical package of SPSS (version 20.0). Since this is an exploratory study, statistical tests were considered significant at the p <0.05 level.

## Results

Table 1 shows the demographic and clinical characteristics of our patients according to their present episode polarity. No statistically significant differences among the four groups were observed as regards age, sex, educational level, marital status, job and financial need.

**Table 1:** Demographic and clinical differences in 150 bipolar heroin addicts according to their present episode polarity

**Demographics**	**(1) Depressive Episode N = 103**	**(2) Hypomanic Episode N = 22**	**(3) Manic Episode N = 5**	**(4) Mixed Episode N = 20**	**Phi**	**p**
	**N (%)**	**N (%)**	**N (%)**	**N (%)**		
Age (M ± sd)	28.81 ± 6.4	28.77 ± 4.7	26.80 ± 5.6	28.45 ± 5.1	0.69	0.55
Gender (males)	63 (61.2)	13 (59.1)	3 (60.0)	12 (60.0)	0.03	0.99
Low Education Level (≤8 yrs)	71 (68.9)	16 (72.7)	4 (80.0)	13 (65.0)	0.57	0.90
Marital status (single)	62 (60.2)	11 (50.0)	3 (60.0)	9 (45.0)	2.05	0.56
Unemployed	48 (46.6)	11 (50.0)	3 (60.0)	8 (40.0)	6.20	0.71
Financial need	20 (19.6)	4 (18.2)	0 (0.0)	7 (35.0)	3.93	0.26
DAH-Q factors						
1. Somatic comorbidity	87 (84.5)	17 (77.3)	4 (80.0)	19 (95.0)	2.65	0.44
2. Altered mental status	103 (100)	22 (100)	5 (100)	20 (100)	-	-
3. Work, major problems	73 (70.9)	13 (59.1)	3 (60.0)	11 (55.0)	2.71	0.43
4. Family, major problems	69 (67.0)	10 (45.5)	2 (40.0)	11 (55.0)	5.02	0.16
5. Romantic involvement, major problems	37 (35.9)	8 (36.4)	1 (20.0)	7 (35.0)	0.54	0.90
6. Social leisure, major problems	60 (58.3)	11 (50.0)	2 (40.0)	8 (40.0)	2.86	0.41
7. Legal problems	53 (51.5)	10 (45.5)	2 (40.0)	7 (35.0)	2.03	0.56
8. Poly-abusers	56 (54.4)	12 (54.5)	4 (80.0)	14 (70.0)	2.80	0.42
9. Past opioid agonist treatment failure(s)	86 (83.5)a	19 (86.9)a	4 (80.0)a	11 (55.0)b	9.15	0.02
10. Associated treatment needed	103 (100)	22 (100)	5 (100)	20 (100)	-	-

Each (a,b) letter indicates a subset of categories “present episode polarity” which portions of the column are not very different at level .05.

Nor were any statistically significant differences observed either among the majority of DAH-RS factors (somatic comorbidity, altered mental status, work, family, romantic involvement, social leisure and legal problems, poly-abusers, associated treatments).

Table 2 shows differences regarding concomitant substance abuse between the four groups of patients.

**Table 2:** Concomitant substance abuse in 150 bipolar heroin addicts according to their present episode polarity

	**(1) Depressive Episode N = 103**	**(2) Hypomanic Episode N = 22**	**(3) Manic Episode N = 5**	**(4) Mixed Episode N = 20**	**Phi**	**p**
	**N (%)**	**N (%)**	**N (%)**	**N (%)**		
Alcohol	50 (48.5)b	8 (38.1)b	1 (20.0)b	15 (75.0)a	0.23	0.044
Anxiolytics and/or Hypnotics	72 (70.6)a	10 (45.5)b	2 (40.0)b	5 (25.0)b	0.34	0.000
Cocaine-amphetamines	19 (18.6)b	16 (72.7)a	5 (100.0)a	13 (65.0)a	0.53	0.000
Cannabinoids	40 (39.6)b	9 (40.9)b	4 (80.0)a	15 (75.0)a	0.27	0.011

Each (a,b) letter indicates a subset of categories “present episode polarity” which portions of the column are not very different at level .05.

No statistically significant differences were observed regarding the abuse of heroin.

Patients with a depressive episode at clinical presentations showed more frequent use of unprescribed anxiolytic-hypnotics. During a hypomanic episode, patients more frequently used cocaine-amphetamines, while, during a manic episode, patients more frequently used cannabis and cocaine-amphetamines. The associated use of alcohol, cocaine-amphetamines and cannabinoids was more frequently encountered during a mixed episode.

## Discussion

As we observed in our sample, patients take anxiolytic-hypnotics, which belong to the class of central nervous system (CNS) depressants, with greater frequency during a depressive episode. They take CNS stimulants (cocaine-amphetamines) at a greater frequency during a hypomanic episode, whereas they tend to take both CNS stimulants and cannabinoids with a greater frequency during a manic episode; lastly, during a mixed episode they take CNS depressants (alcohol), stimulants, and hallucinogens together.

In the case of depressed patients, the use of CNS depressants is consistent with their toxicological status. It should be noted that benzodiazepine use in heroin addicts could be correlated with a condition of opiate dependence improperly compensated by street heroin [38]. From a psychopathological standpoint, depressants may aggravate the slowing of cognitive and physical functions caused by depression, but it remains true that these medications are effective in treating insomnia and anxiety, which are often symptoms of depression. Also, patients may not be seeking an actual ‘lift’ of their depression but be searching for a state of ‘oblivion’ in which the pain of depression is cancelled. In depression, what is seen is not a higher use of stimulant substances, but the use of CNS Depressants that may sometimes relieve some aspects of depression (or may not do so) – a situation that fails to provide support to Khantzian’s hypothesis.

More clearly, Khantzian’s hypothesis does not seem to be supported by the other three kinds of clinical presentations. Patients during a hypomanic, manic or mixed episode, despite experiencing a state of excitement, tend to continue their abuse of psychostimulants, further reinforcing and elevating their mental state. This is consistent with a proposed bipolar-stimulant spectrum where subthreshold bipolar traits are aggravated by stimulant abuse [39].

If we focus on heroin-dependent subjects, the concomitant use of cocaine is reported to be a relevant phenomenon that will determine negative consequences on social adjustment and outcome. When heroin and heroin-cocaine abusers have been compared, a direct relationship has been found between cocaine abuse and the rate of psychiatric disorders, together with correlation with the severity of self-rated psychopathology [40]. We do not know if this lack of awareness of psychopathological symptoms is due to the use of cocaine or to the underlying excitement that sustains cocaine use. Moreover, if cocaine use represents self-enhancement of one's level of hypomania, cyclothymia or hyperthymia, the craving for hypomania is likely to be particularly strong in heroin addicts, whose level of excitement is lowered by heroin use [15]. In addition, cocaine has been reported to induce a higher frequency of mixed states when abused by bipolar patients [17]. This evidence suggests that some bipolar patients, after deciding to use cocaine instead of being excited, may have shifted from a manic or hypomanic to a mixed episode.

During a manic episode, patients show a high level of consumption of stimulants and cannabinoids. In the literature, the abuse of cannabinoids in bipolar patients has been found to induce manic symptoms [24], so it is possible that, in our manic patients, as with cocaine use, they may use cannabinoids to optimize their level of excitement. To date, cannabis use is also considered to be one of the most important risk factors for schizophrenia, thanks to its ability to precipitate or exacerbate psychotic symptoms [41-46]. In line with this assumption, in our sample, cannabis is mainly abused in manic and mixed states that, unlike depressive and hypomanic episodes, are often characterized by the presence of psychotic symptoms. Whatever the causes of the use of cannabis, Khantzian’s hypothesis is not supported in its application to cannabis use. For many subjects, ending cannabis use is difficult to achieve, not only because of prior habits of use, but also because of the attendant psychotic symptoms, including poor insight and judgment, lack of impulse control and cognitive impairment. Most of these subjects are unable to understand that cannabis use is connected with the onset of symptoms [47].

One widely debated issue is whether Khantzian’s hypothesis is a suitable instrument for interpreting alcohol dependence. In examining patients with a mixed episode, we found that, besides their abuse of cocaine-amphetamines and cannabinoids, and in contrast with the other three clinical presentation groups, they often resort to alcohol use. Patients experience their mixed mood as something undesirable and unpleasant, but they still continue to consume substances that tend to preserve their mixed, dysphoric state. Craving for substances and dependence create a loop, a senseless vicious circle in which patients obtain neither satisfaction (mood elation) nor physical benefit (relief). In line with this observation, a past or current alcohol use disorder has proved to raise the likelihood of a switch from depressive to manic, mixed or hypomanic states in patients with bipolar disorder [48]. Nervousness in alcoholic patients has been hypothesized to be the only negative mood state to predict increases in alcohol consumption later in the course of the day. Further examination of this within-person relationship has demonstrated that men were more likely to consume alcohol when nervous than were women, but this association is unrelated to family history of alcoholism, problem drinking patterns, or traits of anxiety and depression. Consistently with the self-medication hypothesis, alcohol consumption has been associated with lower levels of nervousness, but this effect varies in a way dependent on several demographic and clinical variables [49]. Almost one quarter of individuals with mood disorders use alcohol or drugs to relieve symptoms, with the highest prevalence of self-medication in bipolar I disorder. After checking the effects of substance use disorders, self-medication has been associated with higher rates of comorbid anxiety and personality disorders than those found in individuals who do not self-medicate [50]. On the basis of these data we believe that, in the case of alcohol, Khantzian’s hypothesis accounts for anxiety disorders more satisfactorily than mood disorders, although it must be added that it actually explains controlled rather than addictive use. In fact, enduring use, despite the worsening, or the inadequate balance, of symptoms, is inconsistent with a current self-medicating explanation, although that explanation may have been appropriate in a previous stage of controlled use. It should also be remembered that patients were assessed for mood during current drug use, thus ruling out the ambiguity between spontaneous mood swings and substance-induced intoxication. In fact, our patients had been displaying affective dysregulation for some time before being diagnosed, and had been engaged in substance use, which was bound to worsen the affective core of their clinical pictures (i.e. stimulants during excitement and depressants during depression). The hypothesis of symptomatological overlap between temporary substance-related intoxication and mood states is superseded in this way.

After reviewing our data we speculate that, setting aside depressive and mixed episodes, the abuse of substances in hypomanic and manic episodes of bipolar disorder is more probably due to patients’ desire to maintain their current affective state rather than to resolve depressed mood.

These considerations also provide a possible explanation for the fact that bipolar patients tend not to comply with therapy during hypomanic, manic and mixed states [51-53]. In the literature, patients’ lack of compliance with prescribed therapy has been principally associated with their lack of insight into their mental illness [54-56]. We go beyond that in suggesting that, during hypomanic and manic phases, bipolar patients do not comply with prescribed therapies and tend to exacerbate their mental status by means of substances, not only because they are unaware of their mental illness, but also because they somehow live the current episode as a pleasurable and rewarding experience (in cases of addiction to mania).

The obvious limitations of this study are due to the fact that this is a retrospective analysis carried out on a small cohort of patients, rather than a study specifically designed to elucidate this issue. Assessments of the same subject in various different clinical presentations of the natural history of this illness would have provided a better level of information. In addition we must consider the difficulty in determining whether the substance use modifies the mood or the mood state determines the substance used. It is possible that stimulants are seen in those with mania or hypomania because the stimulant produced the mood state. They could have been depressed without it. Lastly, we have no information about the temperament of our subjects. So we cannot exclude the presence of a depressive temperament that is able to moderate the nature of patients’ substance abuse; that would set up the need to modify our hypothesis. One of our earlier findings, however, was that heroin users mainly have a cyclothymic temperament [57].

## Conclusions

In summary, our data do not support Khantzian’s hypothesis, if we exclude the use of CNS depressants during a depressive phase to alleviate anxiety. Also in this case, it can be said that CNS depressants are able to alleviate insomnia and anxiety, but, more generally, may aggravate the slowing of cognitive and physical functions caused by depression. Khantzian’s hypothesis, more evidently, does not seem to be able to account for the use of psychostimulants and cannabis during a hypomanic, manic or mixed state. In fact, the use of these substances definitely leads to the further destabilization of mood. On alcohol use we have to say that alcohol use, in our subjects, is concomitant with a mixed state. Conversely, we would have expected the use of alcohol during hypomanic and manic states to limit the euphoric condition. Lastly, apart from the expected result of a prominent use of CNS stimulants during a depressive phase of bipolar patients, in line with Khantzian’s hypothesis, this study showed that they were used more prominently during hypomanic or manic phases. This apparently supports an alternative hypothesis – that mood elation is a pleasurable and rewarding experience that, in bipolar patients, can be started or prolonged by the use of CNS stimulant drugs.

## Competing interests

There was no conflict of interest.

## Authors’ contributions

IM, MP, AGIM designed the study and wrote the protocol. FR, LR, SB managed the literature searches and analyses. IM undertook the statistical analysis, and all the authors discussed the results. IM, MP, AGIM wrote the first draft of the manuscript. LDO and HA revised the last draft. All the authors contributed to, and have approved, the final manuscript.
